# Teaching & Learning Guide for: Animal Sentience

**DOI:** 10.1111/phc3.12878

**Published:** 2022-10-31

**Authors:** Heather Browning, Jonathan Birch

**Affiliations:** ^1^ London School of Economics and Political Science London UK

## 1 AUTHORS’ INTRODUCTION

Animal sentience, in a broad sense, refers to the capacity of an animal to have feelings with a subjective character. If an animal is sentient, there is *something that it is like* to be that animal. These feelings can include pleasure and pain, but also others such as hunger, curiosity, loneliness, and fear. Animal sentience plays an important role in ethical deliberations, as sentient animals are capable of suffering, a capacity frequently taken to ground moral status. It is also increasingly relevant for policy looking to protect the welfare of animals. Precautionary reasoning can be used to award protection to taxa for which there is some reasonable evidence of sentience, even if this evidence is not definitive. The subjective nature of sentience makes it a difficult scientific target. However, there are an increasing range of indirect indicators that can be used together to infer sentience, including those based on the differences in conscious vs unconscious performance by humans on perceptual and learning tasks, and those based on proposed evolutionary functions of sentience. These indicators can then be used to guide inferences about the distribution of sentience throughout the animal kingdom. There is now wide agreement that there is sufficient evidence to warrant the attribution of sentience to most vertebrates, though some sceptics still question the case for fish. Current work on is also suggestive of sentience in some invertebrates, including cephalopod molluscs and decapod crustaceans.

## 2 AUTHORS RECOMMEND

Birch, J., Burn, C., Schnell, A., Browning, H., & Crump, A. (2021). Review of the Evidence of Sentience in Cephalopod Molluscs and Decapod Crustaceans. https://www.lse.ac.uk/business/consulting/assets/documents/Sentience‐in‐Cephalopod‐Molluscs‐and‐Decapod‐Crustaceans‐Final‐Report‐November‐2021.pdf

This report provides a framework of indicators of sentience for assessing the likelihood of sentience in any given taxa; and an assessment of the literature on in relation to these indicators in cephalopod molluscs and decapod crustaceans.

Duncan, I. J. (2006). The changing concept of animal sentience. Applied Animal Behaviour Science, 100, 11‐19. https://doi.org/10.1016/j.applanim.2006.04.011

Gives a history of the concept of sentience and its use within science and the general public from the 15th Century through to today. Discusses some of the scientific challenges in studying sentience, and where further research is needed.

Godfrey‐Smith, P. (2016). Other minds: The octopus, the sea, and the deep origins of consciousness. New York: Farrar, Strauss and Giroux.

Using stories and examples taken from the author's own experiences while scuba‐diving, this book describes the unique minds of octopuses and traces the possible evolutionary origins of consciousness.

Jones, R. C. (2013). Science, sentience, and animal welfare. Biology & Philosophy, 28, 1‐30. https://doi.org/10.1007/s10539‐012‐9351‐1

This paper discusses the role of sentience in moral status and animal welfare policy, summarising the current research on the sentience of a range of nonhuman animals.

Low, P. (2012, July). The Cambridge declaration on consciousness. Presented at the Francis Crick Memorial Conference, Cambridge, England. https://fcmconference.org/img/CambridgeDeclarationOnConsciousness.pdf

This document represents a foundational shift in the study of animal sentience ‐ a declaration by a group of prominent neuroscientists that the weight of evidence was sufficient to conclude that at least some non‐human animals are conscious.

Nagel, T. (1974). What is it like to be a bat? Philosophical Review, 83, 435‐450.

The classic introduction to the problem of studying the minds of other animals – providing an understanding of what it means to be sentient and the limitations of our intuitive understanding of minds very different from our own.

Proctor, H. S., Carder, G., & Cornish, A. R. (2013). Searching for animal sentience: A systematic review of the scientific literature. Animals, 3(3), 882‐906. https://doi.org/10.3390/ani3030882

Though now slightly out of date, this article provides a thorough review of the literature on animal sentience at the time, showing trends, areas of focus, and knowledge gaps.

## 3 Online Materials

### 3.1 The Foundations of Animal Sentience project

https://www.lse.ac.uk/cpnss/research/ASENT

Website with the latest research outputs of the authors' research project into animal sentience.

### 3.2 Animal Sentience – An Interdisciplinary Journal on Animal Feeling

https://www.wellbeingintlstudiesrepository.org/animsent/

An open‐access journal containing target articles and commentaries on a variety of issues in animal sentience.

### 3.3 Royal Society for the Prevention of Cruelty to Animals – Animal Sentience

https://science.rspca.org.uk/sciencegroup/sentience

Describes the importance of sentience and how to help the welfare of sentient animals, provides links to the outcomes of a 2019 conference on the topic.

## 4 Sample Syllabus

### 4.1 Week 1: Introduction and overview

*What is sentience, and what role does it play in ethics and public policy?*

Readings:

Birch, J. (2017). Animal sentience and the precautionary principle. Animal Sentience, 2(16), 1.
Nagel, T. (1974). What is it like to be a bat? Philosophical Review, 83, 435‐450.
Singer, P. (1979). Practical ethics. Cambridge: Cambridge University Press. – Chapter 3: Equality for animals?

### 4.2 Weeks 2‐3: How to study sentience

*How can scientists gain understanding of sentience, its function, mechanisms, and distribution?*

Readings:

Ben‐Haim, M. S., Dal Monte, O., Fagan, N. A., Dunham, Y., Hassin, R. R., Chang, S. W., & Santos, L. R. (2021). Disentangling perceptual awareness from nonconscious processing in rhesus monkeys (Macaca mulatta). Proceedings of the National Academy of Sciences, 118, e2017543118.
Birch, J. (2020). The search for invertebrate consciousness. Noûs. Advance online publication, https://doi.org/10.1111/nous.12351
Birch, J., Schnell, A. K. & Clayton, N. S. (2020b). Dimensions of animal consciousness. Trends in Cognitive Sciences, 24, 789‐801.
Boly, M., Seth, A.K., Wilke, M., Ingmundson, P., Baars, B., Laureys, S., Edelman, D. and Tsuchiya, N. (2013). Consciousness in humans and non‐human animals: Recent advances and future directions. Frontiers in Psychology, 4, 625.
Nieder, A., Wagener, L., & Rinnert, P. (2020). A neural correlate of sensory consciousness in a corvid bird. Science, 369, 1626‐1629.
Paul, E. S., Sher, S., Tamietto, M., Winkielman, P., & Mendl, M. T. (2020). Towards a comparative science of emotion: Affect and consciousness in humans and animals. Neuroscience & Biobehavioral Reviews, 108, 749–770.

### 4.3 Weeks 4‐5: Distribution of sentience

*Critical discussion of the evidence of sentience across a range of ‘controversial’ taxa, such as fish, cephalopod molluscs, and decapod crustaceans.*

Readings:

Appel, M., & Elwood, R. W. (2009). Motivational trade‐offs and potential pain experience in hermit crabs. Applied Animal Behaviour Science, 119, 120–124.
Birch, J., Burn, C., Schnell, A., Browning, H., & Crump, A. (2021). Review of the Evidence of Sentience in Cephalopod Molluscs and Decapod Crustaceans.https://www.lse.ac.uk/business/consulting/assets/documents/Sentience‐in‐Cephalopod‐Molluscs‐and‐Decapod‐Crustaceans‐Final‐Report‐November‐2021.pdf
Crook, R. J. (2021). Behavioural and neurophysiological evidence suggests affective pain experience in octopus. iScience, 24, 102229.
Key, B. (2016). Why fish do not feel pain. Animal Sentience, 3, 1.
Michel, M. (2019). Fish and microchips: On fish pain and multiple realization. Philosophical Studies, 25, 95‐110.
Sneddon, L. U. (2009). Pain perception in fish: indicators and endpoints. Institute for Laboratory Animal Research Journal, 50(4), 338‐342.

## 5 FOCUS QUESTIONS

1. What is sentience?
2. What is the ethical significance of sentience?
3. How should sentience guide public policy?
4. How can we study sentience?
5. Which animals are sentient?

## 6 SEMINAR/PROJECT IDEA

Practical assignment: Assessing the evidence for sentience.

Choose one of the following taxa: insects, gastropod molluscs, or spiders. Argue for or against sentience in this taxonomic group, providing reasons for your judgement (including empirical evidence where available).

